# Weight Change and Risk of Atherosclerosis Measured by Carotid Intima–Media Thickness (cIMT) from a Prospective Cohort—Analysis of the First-Wave Follow-Up Data of the Canadian Longitudinal Study on Aging (CLSA)

**DOI:** 10.3390/jcdd10100435

**Published:** 2023-10-19

**Authors:** Jian Liu, Newman Siu Kwan Sze, Miya Narushima, Deborah O’Leary

**Affiliations:** Department of Health Sciences, Brock University, St. Catharines, ON L2S 3A1, Canada; nsze@brocku.ca (N.S.K.S.); mnarushima@brocku.ca (M.N.); doleary@brocku.ca (D.O.)

**Keywords:** CLSA, cIMT, weight change, prospective cohort, atherosclerosis

## Abstract

To explore impact of weight change (WC) on risk of atherosclerosis measured by cIMT, 20,700 participants from the CLSA follow-up were included in analysis. WC was defined as the difference of weight measured at follow-up and baseline, then quartered into four groups (Q1–Q4). cIMT > 1.0 mm was defined as high risk for atherosclerosis. Adjusted odds ratio (OR (95% CI)) from logistic regression models were used to evaluate the association between WC and risk of atherosclerosis. At follow-up, participants had gained 0.118 kg weight, on average, and 16.4% of them were at high risk for atherosclerosis. The mean levels of cIMT were comparable between participants from Q1 to Q4. Compared to Q2 (reference), the ORs (95% CI) were 1.00 (0.86, 1.15), 1.19 (1.03,1.38), and 1.25 (1.08,1.45) for Q1, Q3, and Q4, respectively. A similar pattern was observed when analyses were conducted for ages < 65 vs. 65+ separately, but it was weaker for those aged 65+. Results from the jointed distribution analyses indicated that moderate weight loss might increase risk for atherosclerosis among participants with obese BMI at baseline, but not for those with cardiovascular event status at baseline. Weight gain, however, would increase risk for atherosclerosis regardless of cardiovascular event status, or overweight/obese BMI at baseline.

## 1. Introduction

Carotid intima–media thickness (cIMT) as a surrogate of atherosclerosis upon cardiovascular risk prediction and treatment evaluation has been extensively studied [1]. Evidence suggests that cIMT modestly improves cardiovascular risk assessment in addition to traditional cardiovascular risk factors [2], and it may be a useful indicator for monitoring cardiovascular disease (CVD) risk progress in clinical practice [3]. Weight changes over a short time period, either loss or gain, among community-dwelling older adults are common and most of them are unintentional [4]. Results from the Korean Longitudinal Study of Ageing suggest that a weight loss of 5 kg or more within two years significantly increases risk for all-cause mortality [5]. On the other hand, obesity, defined as an excess of body fat mass, is extensively associated with many chronic diseases, in particular CVD [6]; as its prevalence has constantly increased globally, it has become a serious public health concern [7]. Longitudinal studies reveal that childhood body mass index (BMI) is positively related to cIMT in young or middle-aged adults [8,9]. Studies conducted among older adults or menopausal women also showed that high BMI is associated with elevated levels of cIMT [10,11].

Weight loss for people suffering from obesity is considered an effective approach to lowering CVD risk. For example, a meta-analysis conducted with small studies having examined weight loss through surgical, diet/exercise, or pharmacological interventions among obese individuals found that weight loss was associated with reduction in cIMT [12]. However, a generously sized randomized clinical trial conducted among overweight or obese adults with type 2 diabetes did not show that medical weight loss could lead to a reduction in incidence of coronary artery disease [13]. Therefore, there are still debates on how to apply cIMT in the prediction of CVD, particularly among older adults, as they often have chronic conditions such as high blood pressure, glucose, and/or dyslipidemia [2,14,15]. In addition, there are no studies that have examined the relationship between cIMT and weight change among middle-aged and older adults. Thus, using the first follow-up of the Canadian Longitudinal Study on Aging (CLSA), we aim to assess the relationship between weight change and atherosclerosis measured by cIMT.

## 2. Materials and Methods

### 2.1. Participants from the Canadian Longitudinal Study on Aging (CLSA)

Approximately 51,000 men and women aged 45–85 across Canada (mean age 60 years) were enrolled in the baseline (2011–2015) of two subcohorts of the CLSA, i.e., tracking and comprehensive. Participants from the comprehensive cohort were randomly selected from within a 25–50 km radius of one of the eleven data collection sites (DCSs) in seven Canadian provinces and completed in-person home interviews and underwent physical assessments at a DCS. Participants from the tracking cohort were excluded because they had no measurements of cIMT and weight for the analysis. There were 30,097 participants from the comprehensive cohort at the baseline, 27,765 of them were followed at the first wave of the follow-up (2015–2018), and a total of 20,700 men and women who had complete measurements of weight and cIMT were included in this study. Detailed information of the CLSA regarding study design and data collection at baseline and first wave of follow-up can be found elsewhere [16,17].

### 2.2. Measurement of cIMT

The cIMT measurement was conducted at baseline as well as first-wave follow-up at the DCS following the standard operating procedure [18]. All participants from the comprehensive cohort who were able to stand without the assistance of another person were eligible for the measurement. The participant was required to lie on the exam bed for at least five minutes prior to measurement. Carotid artery images were obtained using a 12 MHz linear array probe attached to a high-resolution ultrasound system (GE VIVIDi) with concurrent electrocardiogram gating at all testing sites. Average IMT, maximum IMT, and minimum IMT from both right and left carotid arteries were determined during diastole. The average cIMT value is the most common measurement used in cardiovascular risk prediction studies [3]; thus, in this study, we focused on the relationship between weight change and average IMT from both the right and left carotid artery at the first wave of follow-up. Based on whether the cIMT values were larger than 1.0 mm, which is suggestive of plaque, a sign for high-risk of atherosclerosis [2], participants were also grouped into two categories: those with either the right or left cIMT average > 1.0 mm, or those with cIMT ≤ 1.0 mm.

### 2.3. Weight Change

Weight values at both baseline and follow-up were measured at the DCS by trained research assistants using the 140–10 Healthweight Digital Physician Scale and recorded to the nearest 0.1 kg according to the standard arthrometric operating procedure [19]. The weight change was defined as the difference of the weight values measured at follow-up and baseline. All participants were further categorized into four groups based on the distribution of weight change quartile cutoffs, i.e., Q1: <−2.25; Q2: −2.25~0.10; Q3: 0.10~2.35, Q4: ≥2.35 (kg).

### 2.4. Measurements of Demographic Variables and Other Cardiovascular Risk Factors at Baseline

Demographic variables: age (years), sex (male vs. female), education (bachelor’s degree or above vs. other), marital status (married or living with a partner vs. other), and country of birth (Canada vs. other).

Cardiovascular risk factors: (a) **Variables derived from the in-person home interview**: current smoking (yes vs. no), alcohol (having drank alcohol during the past 12 months: yes vs. no), general health (good or excellent vs. other), CESD10 (Center for Epidemiological Studies Depression Scale 10 questions version, a score to detect current depressive symptomatology), and cardiovascular events (including historical heart disease, stroke, diabetes, and/or hypertension: yes vs. no). (b) **Variables derived from laboratory and physical examination**: total cholesterol (mol/L), high-density lipoprotein (HDL) cholesterol (mol/L), non-HDL cholesterol (mol/L), systolic blood pressure (SBP mmHg), diastolic blood pressure (DBP mmHg), body mass index (BMI kg/m^2^), waist circumference (cm), weight (kg), and height (m).

The demographic variables, as well as those related to lifestyle and general health, were collected through a questionnaire survey conducted during in-person home interviews. Blood pressure measurements and physical examination were conducted at the DCS by trained research assistants. Non-fasting venipuncture blood samples were collected for lipid profiles at the DCS.

### 2.5. Statistical Analysis

All analyses were conducted using RStudio (R version 4.2.2). Statistical significance was set at two-sided α < 0.05. Mean SE (standard error) was used to present central tendency and variability for continuous variables and percentage proportions were used for categorical variables. For the univariate analysis, one-way ANOVA (analysis of variance) was used for continuous variables and chi-square test for categorical variables.

There were significant differences in age and sex among participants in weight change quartiles; therefore, general linear models were used to obtain the age- and sex-adjusted mean levels of cIMT and logistic regression was used to obtain the age- and sex-adjusted prevalence of cIMT > 1.0 mm at follow-up.

**Figure 1:**
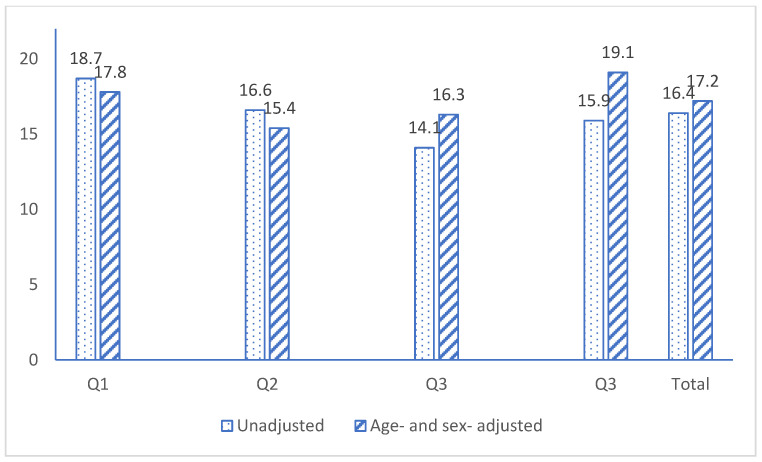
Proportion (%) of cIMT > 1.0 mm by quartile of weight change at the first follow-up.

Four logistic regression models were created to examine the risk association of cIMT > 1.0 mm at follow-up for weight change and weight change values in the second quartile (Q2) served as the reference group because it had the lowest age- and sex-adjusted prevalence of cIMT > 1.0 mm (see Figure 1). Covariates included in the models were those statistically significant or borderline significant in the univariate analysis. Odds ratios (OR 95% confidence interval (CI)) were used as the evaluation indicator for the risk association. In model one, variables of age, sex, marital status, education, and birth country were adjusted. In model two, we further adjusted for health- and lifestyle-related variables, which included self-reported general health, current smoking, and alcohol intake. In model three, we further adjusted for CVD-related risk factors, including SBP, HDL cholesterol, standing height, and right cIMT at baseline (since more participants had right cIMT value at baseline). Finally, in model four, we further adjusted for BMI and cardiovascular events reported at baseline.

Since weight loss is an important risk factor of all-cause mortality for older people, and they are also more likely to lose weight [20], we further evaluated the risk association of cIMT > 1.0 mm with weight change by age group; participants were categorized into under 65 years or 65 years and above. In addition, we also examined the impact of baseline BMI or reported cardiovascular events on the observed risk association. Based on the obesity criteria, participants were categorized into (a) normal weight BMI < 25 kg/m^2^, (b) overweight BMI 25~29.9 kg/m^2^, and obese BMI> 30 kg/m^2^. With weight change quartiles, we created 12 groups (3 × 4). In the risk association analysis, participants who were of normal weight (BMI < 25 kg/m^2^) and had experienced a change in weight in the second quartile (Q2) served as the reference group. Based on the cardiovascular events reported at baseline (yes vs. no) and weight change quartiles, we created eight groups (2 × 4), and participants who did not have cardiovascular events reported at baseline and weight change in the second quartile (Q2) were used as the reference in the risk association analysis. All covariates included in these models were like those in model 4 with some necessary modification, e.g., variable cardiovascular events would be dropped off when analyzing the interaction effect with weight change quartiles. To ensure generalizability, the trimmed inflation weight was used for calculation means (SEs) and proportions and analytic weight was used to count the impact of sampling strategy when examining relationships between variables [21].

## 3. Results

At follow-up, participants’ weight, on average, increased 0.118 kg (Q1: −5.79 kg; Q2: −0.95 kg; Q3: 1.18 kg; Q4: 5.29 kg); if cIMT > 1.0 mm was used to define the elevated risk for atherosclerosis, 16.4% of participants fell into this category at follow-up.

### 3.1. Summaries of the Descriptive Statistics

Table 1 shows the descriptive statistics for selected demographics and other cardiovascular risk factors by weight change quartile. Participants in Q1 differed statistically from people in the other weight change quartiles (Q2–Q4) in certain demographic and lifestyle variables including having the highest proportions of males (51.2%) and current smokers (12.3%), but the lowest proportions of married or living with a partner (72.7%), bachelor’s degree education (40.6%), self-reported good or excellent general health (89.2%), and alcohol intake during the past year (84.8%). The proportion of birth country was comparable with other quartiles. They had a poor cardiovascular profile, their total cholesterol mean level was comparable to other weight change quartiles, but they had the lowest mean level of HDL, highest mean levels of blood pressures, non-HDL cholesterol (though not significant), and measurements related to body compositions (i.e., BMI, waist circumference, weight, but not height); in addition, over half of them reported cardiovascular events at baseline. Interestingly, people in Q4 weight change group were much younger than other groups and they had a comparable CVD risk profile to those in Q1 (Table 1).

The overall prevalence of cIMT > 1.0 mm was 17.2% after adjusting for age and sex, which was a little higher than the unadjusted prevalence (16.4%). The unadjusted prevalence rates of cIMT > 1.0 mm for people in Q1 to Q4 were 18.7%, 16.6%, 14.1%, and 15.9%, respectively (Figure 1); however, after adjusting for age and male sex, the highest prevalence rate was found among individuals in Q4 (19.1%), participants in Q1 had the second highest (17.8%), and people in Q2 had the lowest adjusted prevalence (15.4%).

### 3.2. Adjusted ORs of cIMT > 1.0 mm for Weight Change in Different Situations

Table 2 shows the adjusted ORs of cIMT > 1.0 mm for weight change in quartiles at the follow-up. After adjusting for age, sex, marital status, education, and birth country (model 1), the ORs (95% CIs) were 1.14 (1.01,129), 1.09 (0.96, 1.23), and 1.29 (1.14, 1.46) for those in Q1, Q3, and Q4, respectively. Further adjusted for variables related to general health and lifestyle (model 2), variables related to cardiovascular risk factors (model 3), and BMI and CVD events reported at baseline (model 4), the OR (95% CI) for Q4 did not change much (1.25 [1.08, 1.45]), however, the OR for Q1 (1.00 [0.86, 1.15]) and Q3 (1.19 [1.03, 1.38]) did change.

Table 3 presents the unadjusted cIMT mean, prevalence of cIMT > 1.0 mm, and adjusted ORs (95% CIs) for weight change quartiles by age group. The overall cIMT means in each age group were similar to what was observed, left cIMT slightly higher than right cIMT, but the mean levels for participants aged 65 or above were significantly higher than for those aged less than 65, i.e., age < 65 vs. 65+, right cIMT (mm): 0.720 vs. 0.853; left cIMT (mm): 0.749 vs. 0.874. Interestingly, there was an approximately 0.13 mm difference between those aged < 65 and 65+ years in cIMT means, but the unadjusted prevalence of cIMT > 1.0 mm for people aged 65 years or above was almost three times higher than for those aged less than 65 (30.0% vs. 11.0%). Using the same covariates as in model 4 and the reference group (Q2), the ORs (95% CIs) were 1.20 (0.98, 1.48), 1.36 (1.11, 1.66), and 1.41 (1.17, 1.72) for weight change quartiles, Q1, Q3, and Q4, among those aged less than 65, respectively, and 0.98 (0.81,1.15), 1.09 (0.91, 1.30), and 1.21 (1.00, 1.48) for weight change quartiles, Q1, Q3 and Q4, among those aged 65 or above, respectively.

**Figure 2:**
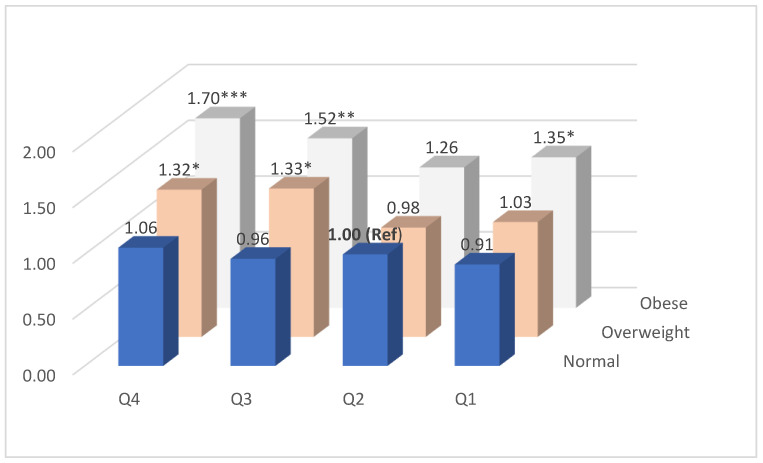
Adjusted OR§ of cIMT > 1.0 mm at follow-up by weight change quartile and obesity status at baseline. Adjusted for age, sex, marriage, education, birth country, general health, current smoking, alcohol drinking, SBP, HDL-c, CVD status; right side cIMT; and height measured at baseline. * *p* < 0.05, ** *p* < 0.001,*** *p* < 0.0001.

The adjusted ORs for weight change quartiles at follow-up and obesity status at baseline are shown in Figure 2. Compared to people who were of normal weight at baseline but with a moderate weight change (Q2) at follow-up, participants with normal weight at baseline but with either weight loss (Q1) or gain (Q3 and Q4) at follow-up had a similar risk association, and this was also true for people who were overweight at baseline but with weight loss (Q1) or moderate weight change (Q2) at follow-up, while for those who were overweight at baseline but with weight gain (Q3 and Q4) at follow-up, there was an over 30% increase in OR for cIMT > 1.0 mm. For people who were obese at baseline regardless of either weight loss (Q1), moderate weight change (Q2), or weight gain (Q3 and Q4) at follow-up, they all had an increased OR of cIMT > 1.0 mm, i.e., 1.35 for Q1, 1.26 for Q2, 1.52 for Q3, and 1.70 for Q4, respectively, and all ORs except for those with moderate weight change (Q2) were statistically significant (*p* < 0.05).

**Figure 3:**
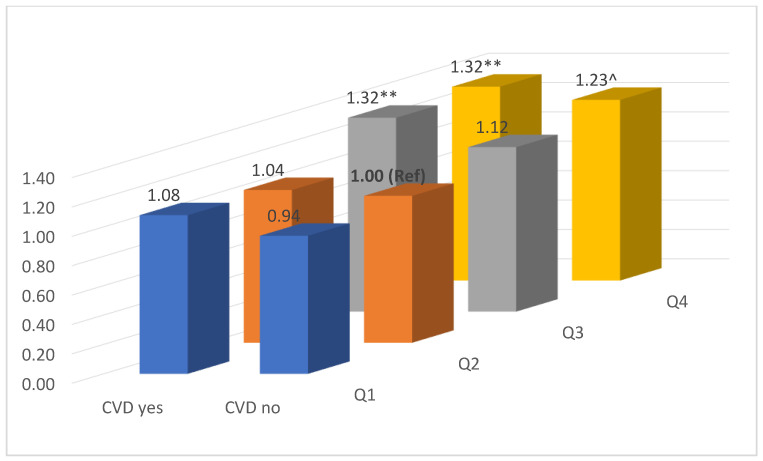
Adjusted OR§ of cIMT > 1.0 mm at follow-up by weight change in quartiles and CVD status at baseline. ** *p* < 0.01, ^ *p* = 0.052. Adjusted for age, sex, marriage, education, birth country, general health, current smoking, alcohol drinking, SBP, HDL-c, height, right side cIMT, and BMI measured at baseline.

The adjusted ORs for weight change in quartiles at follow-up and reported CVD events at baseline are shown in Figure 3. Compared to people with moderate weight change (Q2) at follow-up but without cardiovascular events at baseline, those who lost weight (Q1) regardless of their CVD status had a similar OR for cIMT > 1.0 mm. A similar result was also observed for participants with moderate weight change at follow-up (Q2) but with CVD events at baseline. Compared to the reference group, people with weight gain (Q3 and Q4) all had an increased OR for cIMT > 1.0 mm; however, only for people who had reported CVD events at baseline was there statistical significance (OR = 1.32, *p* < 0.01), while for people without cardiovascular events, the ORs were 1.12 for Q3 and 1.23 for Q4, but only Q4 was borderline significant (*p* = 0.052).

## 4. Discussion

Using the data of the first CLSA follow-up, we examined the relationship between cIMT and weight change in middle-age and older Canadian adults. From that study, we observed that people with weight loss did not improve either their mean cIMT (see Supplementary Figures S1 and S2) or the odds of cIMT > 1.0 mm; moreover, those who were obese at baseline but did lose weight at follow-up had an increased risk for atherosclerosis. For those who gained weight, their cIMT mean at follow-up was comparable to those who lost weight, but they had a significantly increased risk for atherosclerosis; this was more apparent among people aged less than 65 or having had a CVD event at baseline.

Results from previous studies indicate that cIMT values were positively associated with BMI and age [8,9,10,11,22]. In this study, we also observed positive correlations between cIMT levels at follow-up and BMI or age at baseline, though those correlations were weak (Appendix A). However, we did not observe that weight loss for obese people reduced their cIMT, which was suggested by the intervention studies in which diet, exercise, surgery, and/or pharmacologic methods were used to lower weight [12]. There were no statistical differences in mean levels of cIMT at follow-up for people between different weight change quartiles, and this suggests that weight change, either gain or loss, did not affect their mean cIMT. Additionally, the overall adjusted OR for those with weight loss (Q1) was similar to the reference group (Q2), indicating that the prevalence of cIMT > 1.0 mm was comparable. However, we observed increased odds of cIMT > 1.0 mm for people who were obese at baseline but lost weight at follow-up. This could be due to the fact that the CLSA is an observational cohort study, making it difficult to evaluate the relationship between cIMT and weight loss since unintentional weight loss is different from intentional loss. Unintentional weight loss is usually a sign of poor health for old people [23,24]. In fact, the average weight loss for those who were obese at baseline was only 5.6 kg over 36 months follow-up in this study, compared to 16 kg weight loss, on average, over 20 months for obese people from Skilton’s meta-analysis of intervention studies [12]. Therefore, the weight loss might be too small to influence the progress of cIMT, even if their weight loss was intentional. Furthermore, people from the intervention studies highlighted by Skilton et al. [12] were much younger compared to the participants of the CLSA (the mean age of eight of nine studies in that meta-analysis was 48 years or less vs. the average age of 60 at baseline for the CLSA). Since atherosclerosis has a long asymptomatic phase of development and it usually begins early in life, often during childhood [25], its reverse progress would be more challenging for older individuals in comparison to younger individuals.

We observed that those who gained weight at follow-up had an increased risk for atherosclerosis even if their cIMT mean levels were comparable between weight change quartiles. Although the prevalence of cIMT > 1.0 mm at follow-up was much higher in those aged 65 or above than those less than 65, the risk association was much stronger in people aged less than 65. Results from a systematic review by van den Munckhof and colleagues [26] suggest that cIMT is linearly associated with age in the general population, but association between the occurrence of cardiovascular events and age is not linear, demonstrating a sudden increase in event rate after 60 years of age [27]. In fact, our results do not contradict what previous studies have observed. Since atherosclerosis starts in childhood and develops with age, the entire process appears as linear with age, but middle age (45–65) would be the critical stage to observe people whose atherosclerosis risk is reaching a point of higher CVD likelihood, especially when gaining too much weight. Therefore, we may see the odd phenoxenium that overall, middle-aged adults had a lower prevalence of atherosclerosis (cIMT > 1.0 mm) but presented with a significantly increased risk for atherosclerosis when having gained weight, while old-aged adults seemed opposite, having a higher prevalence of atherosclerosis, but the increased risk was not impressive when exhibiting similar weight gain because the differences of risk for atherosclerosis were minimal among old-aged people within different weight change groups. Although medical weight loss among people who are overweight or obese does not reduce the incidence of cardiovascular events [13], our results indicate that weight gain was indeed a contributing factor for increasing atherosclerotic risk (cIMT > 1.0 mm), particularly for people having had cardiovascular events at baseline. We did not find an interaction between sex and weight change regarding the risk of atherosclerosis, though women are generally more likely to lose weight (results not shown).

One limitation of the current study when interpreting the results is that approximately 25% of the cohort’s participants were excluded from the analysis due to either missing measured weight or cIMT at follow-up. Compared to participants who were included in this study, they were older, and more likely to be male and with cardiovascular events at baseline; therefore, the results of this study may not be well generalizable to the general population, particularly for people aged 65 or above. Nevertheless, the large sample size, population-based sampling design and weighting, and adjustment of many well-known covariates can be considered the strengths of this study.

## 5. Conclusions

In conclusion, moderate weight loss observed among middle and/or older adults from this observational cohort does not demonstrate an improvement in cIMT. Nevertheless, weight gain was associated with increased risk for atherosclerosis, particularly among middle-aged adults, people with cardiovascular events, and/or those who were overweight/obese at baseline. Thus, these factors are important to consider when developing efficient prevention and promotion strategies for lowering CVD risk among high-risk populations.

## Figures and Tables

**Table 1:** Baseline selected characteristics by weight change quartile in the first follow-up of CLSA.

	Weight Change Quartiles (kg)				
	<−2.25	~0.10	~2.35	≥2.35	*p*-Value
*n*	4967	5336	5263	5134	
Age (yrs. mean (SE))	60.9 (0.3)	61.0 (0.3)	59.0 (0.3)	56.6 (0.2)	***
Male (%)	51.2	46.1	46.5	48.4	^
Married (%)	72.7	75.4	79.4	75.6	***
Country of birth as Canada (%)	82.0	78.2	80.4	83.7	**
Bachelor’s degree received (%)	40.6	44.3	45.5	41.6	***
General health^ (%)	89.2	92.0	93.5	90.6	***
Current smoking (%)	12.3	8.2	8.8	12.1	***
Alcohol (%)	84.8	87.9	87.7	86.9	***
CESD10^ (mean (SE))	5.83 (0.14)	5.21 (0.14)	5.12 (0.14)	5.67 (0.12)	ns
Total cholesterol (mol/L, mean (SE))	5.16 (0.03)	5.22 (0.03)	5.23 (0.03)	5.16 (0.03)	ns
HDL cholesterol (mol/L, mean (SE))	1.42 (0.01)	1.52 (0.01)	1.52 (0.01)	1.46 (0.01)	***
Non-HDL cholesterol (mol/L, mean (SE))	3.74 (0.03)	3.70 (0.03)	3.71 (0.03)	3.70 (0.03)	ns
SBP (mmHg, mean (SE))	122.9 (0.6)	120.1 (0.4)	118.3 (0.4)	118.9 (0.4)	***
DBP (mmHg, mean (SE))	75.9 (0.3)	74.5 (0.3)	74.3 (0.2)	75.5 (0.3)	*
BMI (kg/m^2^, mean (SE))	29.7 (0.2)	27.1 (0.1)	26.9 (0.1)	28.6 (0.2)	***
Waist circumference (cm, mean (SE))	98.7 (0.5)	91.3 (0.4)	90.7 (0.4)	94.7 (0.5)	***
Height (m, mean (SE))	1.691 (0.003)	1.675 (0.003)	1.680 (0.003)	1.693 (0.003)	*
Weight (kg, mean (SE))	85.1 (0.6)	76.2 (0.4)	76.2 (0.4)	82.2 (0.5)	***
Weight change at the first follow-up (kg, mean (SE))	−5.79 (0.12)	−0.95 (0.02)	1.18 (0.02)	5.29 (0.09)	***
Cardiovascular diseases (%)	50.7	40.6	38.4	42.2	***

CESD10: Center for Epidemiological Studies Depression Scale 10 questions version; SBP: systolic blood pressure; DBP: diastolic blood pressure; BMI: body mass index. *** *p* < 0.0001, ** *p* < 0.01, * *p* < 0.05, ^ *p* < 0.10, ns *p* > 0.10.

**Table 2:** Adjusted ORs of cIMT > 1.0 mm for weight change quartiles at the first follow-up, CLSA.

Weight Change at the First Follow-Up								
		Q1		Q2 (Ref)	Q3		Q4	
		OR	95% CI	OR	OR	95% CI	OR	95% CI
Model 1	*n* = 17,828	1.14	(1.01, 129)	1.00	1.09	(0.96, 1.23)	1.29	(1.14, 1.46)
Model 2	*n* = 17,458	1.12	(0.98, 1.27)	1.00	1.09	(0.96, 1.23)	1.28	(1.13, 1.46)
Model 3	*n* = 13,731	1.07	(0.92, 1.23)	1.00	1.19	(1.03, 1.37)	1.31	(1.13, 1.51)
Model 4	*n* = 13,715	1.00	(0.86, 1.15)	1.00	1.19	(1.03, 1.38)	1.25	(1.08, 1.45)

cIMT: carotid intima media thickness. Model 1: adjusted for age, sex, marriage, education, and country of birth. Model 2: further adjusted for general health, current smoking, and alcohol consumption. Model 3: further adjusted for systolic blood pressure, HDL cholesterol, right side cIMT, and height measured at baseline. Model 4: further adjusted for BMI and CVD status at baseline.

**Table 3:** Statistics of cIMT for weight change quartiles at follow-up by age group, CLSA.

	Weight Change Quartile at Follow-Up				
	Q1	Q2 (Ref)	Q3	Q4	Total
Age					
**<65 years (*n* = 8718)**					
Right cIMT (mm, mean (SE))	0.729 (0.005)	0.716 (0.005)	0.715 (0.005)	0.718 (0.005)	0.720 (0.005)
Left cIMT (mm, mean (SE))	0.751 (0.005)	0.746 (0.006)	0.744 (0.005)	0.749 (0.005)	0.749 (0.005)
cIMT > 1.0 mm (%)	11.6	9.6	9.3	12.3	11.0
OR^ (95% CI)	1.20 (0.98, 1.48)	1.00	1.36 (1.11, 1.66)	1.41 (1.17, 1.72)	
**65 + years (*n* = 4977)**					
Right cIMT (mm, mean (SE))	0.867 (0.008)	0.849 (0.007)	0.838 (0.007)	0.861 (0.010)	0.853 (0.008)
Left cIMT (mm, mean (SE))	0.886 (0.007)	0.872 (0.008)	0.868 (0.007)	0.870 (0.010)	0.874 (0.008)
cIMT > 1.0 mm (%)	32.9	29.7	26.7	30.9	30.0
OR (95% CI)	0.98 (0.81, 1.15)	1.00	1.09 (0.91, 1.30)	1.21 (1.00, 1.48)	

cIMT: carotid intima media thickness; mean (SE): raw mean (standard error); %: unadjusted prevalence; OR (95% CI): adjusted odds ratio (95% confidence interval), same as model 4 in Table 2.

## Data Availability

The CLSA data are available from the Canadian Longitudinal Study on Aging (www.clsa-elcv.ca) for researchers who meet the criteria for access to de-identified CLSA data. (The data used in this analysis are accessed on 13 April 2021.)

## Supplementary Materials

The following supporting information can be downloaded at: https://www.mdpi.com/2308-3425/10/10/435/s1, Figure S1: Unadjusted mean levels of cIMT (mm) by weight change quartile at the follow-up; Figure S2: Age and sex adjusted mean levels of cIMT (mm) by quartile of weight change at the follow-up.
