# Correction: Liu et al. Weight Change and Risk of Atherosclerosis Measured by Carotid Intima–Media Thickness (cIMT) from a Prospective Cohort—Analysis of the First-Wave Follow-Up Data of the Canadian Longitudinal Study on Aging (CLSA). *J. Cardiovasc. Dev. Dis.* 2023, *10*, 435

**DOI:** 10.3390/jcdd11070196

**Published:** 2024-06-28

**Authors:** Jian Liu, Newman Siu Kwan Sze, Miya Narushima, Deborah O’Leary

**Affiliations:** Department of Health Sciences, Brock University, St. Catharines, ON L2S 3A1, Canada; nsze@brocku.ca (N.S.K.S.); mnarushima@brocku.ca (M.N.); doleary@brocku.ca (D.O.)

There was missing information in the back matter of the original publication [1].

A correction has been made to the Data Availability Statement, Acknowledgments, and Conflicts of Interest sections, as follows:

**Data Availability Statement:** The CLSA data are available from the Canadian Longitudinal Study on Aging (www.clsa-elcv.ca) for researchers who meet the criteria for access to de-identified CLSA data. (The data used in this analysis are accessed on 13 April 2021.)
**Acknowledgments:** This research was made possible using the data/biospecimens collected by the Canadian Longitudinal Study on Aging (CLSA). Funding for the CLSA is provided by the Government of Canada through the Canadian Institutes of Health Research (CIHR) under grant reference LSA 94473 and the Canada Foundation for Innovation as well as the following provinces: Newfoundland, Nova Scotia, Quebec, Ontario, Manitoba, Alberta, and British Columbia. This research was conducted using the CLSA Baseline Comprehensive Dataset CoP5 and the CLSA First Follow-up Dataset CoP3 under “Application Number 2010012”. The CLSA is led by Parminder Raina, Christina Wolfson, and Susan Kirkland.
**Conflicts of Interest:** The authors declare no conflicts of interest. The opinions expressed in this manuscript are the authors’ own and do not reflect the views of the Canadian Longitudinal Study on Aging.

The authors state that the scientific conclusions are unaffected. This correction was approved by the Academic Editor. The original publication has also been updated.
