# Supplementary material for: Weight Change and Risk of Atherosclerosis Measured by Carotid Intima–Media Thickness (cIMT) from a Prospective Cohort—Analysis of the First-Wave Follow-Up Data of the Canadian Longitudinal Study on Aging (CLSA)
Source: J Cardiovasc Dev Dis. 2023 Oct 19;10(10):435. doi: 10.3390/jcdd10100435 (PMC10607020; doi:10.3390/jcdd10100435)

Supplementary Figure S1. Unadjusted mean levels of cIMT (mm) by weight change quartile at the follow-up.

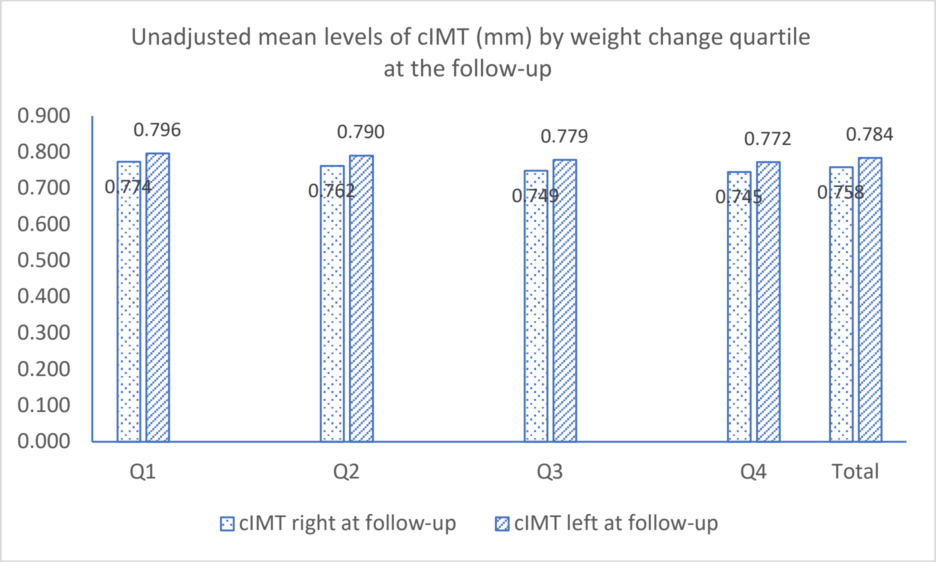

Supplementary Figure S2. Age and sex adjusted mean levels of cIMT (mm) by quartile of weight change at the follow-up.

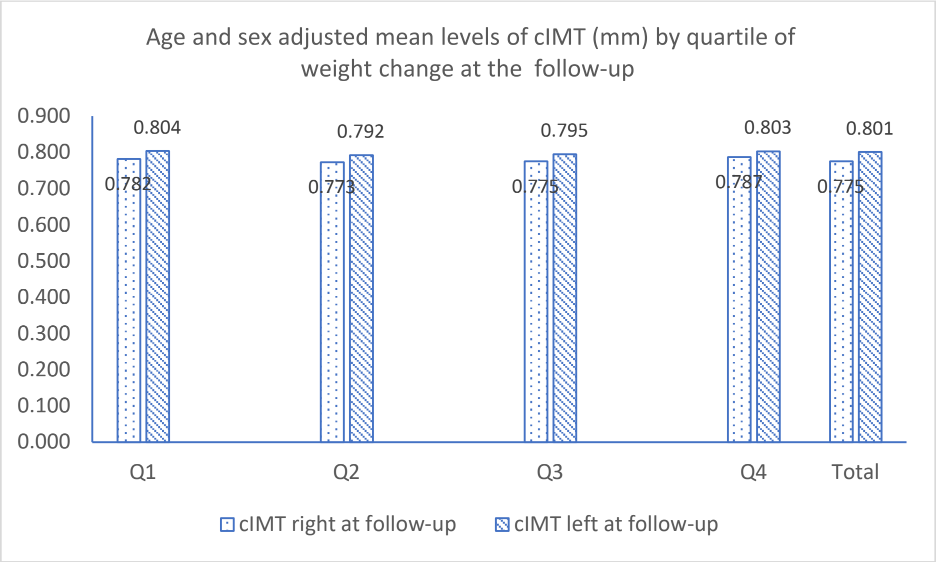

Supplement: Supplementary file 1 [file jcdd-10-00435-s001.zip › jcdd-2642523-supplementary.pdf]
